# Supplementary material for: Risk of stroke in male and female patients with atrial fibrillation in a nationwide cohort
Source: Nat Commun. 2024 Aug 7;15:6728. doi: 10.1038/s41467-024-51193-0 (PMC11306344; doi:10.1038/s41467-024-51193-0)

Supplemental Table 1: ICD10 codes and ATC-codes

	International Classification of Diseases 10th revision (ICD-10) code	Anatomical Therapeutic Chemical (ATC) code
**Clinical characteristics**		
Congestive heart failure	I11.0 I13.0 I13.2 I42.0 I50	CO3C
Left ventricular dysfunction	I50.1 I50.9	
Hypertension		See specified definition*
Diabetes mellitus	E10.0 E10.1 E10.9 E11.0 E11.1 E11.9	A10
Ischemic stroke	I63 I64	
Systemic embolism	I74	
Transient ischemic attack	G45	
Aortic plaque	I70.0	
Peripheral arterial disease	I70.2-I70.9 I71 I73.9 I74	
Myocardial infarction	I21-I23	
Chronic kidney disease	I12 I13 N00-N05 N07 N11 N14 N17-N19 Q61	
Liver disease	B15.0 B16.0 B16.2 B19.0 K70.4 K72 K76.6 I85	
Bleeding event	I850 I864A K226 K228F K250 K252 K254 K256 K260 K262 K264 K266 K270 K272 K274 K276 K280 K282 K284 K286 K290 K298A K625 K638B K638C K661 K838F K868G K920 K921 K922 I60 I61 I62 I690 I691 I692 S063C S064 S065 S066 S068B S068D S141C S141D S141E S241D S241E S241F S341D S341E S341F E078B E274B G951A I312 I319A I230 J942 M250 R04 S259A	

Alcohol intake	E22.4 E52.9A F10 G31.2 G62.1 G72.1 I42.6 K29.2 K70 K86.0 L27.8A O35.4M T51 Z71.4 Z72.1	
Atrial fibrillation	I48	
Alcohol abuse	E244 E529A F10 G312 G621 G721 I426 K292 K70 K860 L278A O354 T51 Z714 Z721	
Cancer diagnosis	C	
COPD	J40 J41 J42 J43 J44 J45 J46 J47 J60 J61 J62 J63 J64 J65 J67 J684 J701 J703 J841 J920 J921 J982 J983	
Ischemic heart disease	I20 I21 I22 I23 I24 I25	
CABG procedure	KFNA KFNC KFND KFNE	
PCI procedure	KFNG	
**Medication information**		
Dabigatran		B01AE07
Rivaroxaban		B01AF01
Apixaban		B01AF02
Edoxaban		B01AF03
Warfarin		B01AA03
Aspirin		B01AC06
Clopidogrel		
Beta-blockers		C07

Calcium channel blockers		C07F C08 C09BB C09DB
Renin-angiotensin system inhibitors (ACEi/ARBs)		C09
Loop diuretics		C03C
Statin		C10
NSAID		M01AA M01AB M01AC M01AE M01AG M01AH M01AX01
Non-loop diuretics		C02DA C02L C03A C03B C03D C03EA C03X C07C C07D C08G C09BA C09DA C09XA52
PGP inhibitors		J02AB02 J02AC02 L04AD02 L04AD01 C08DA01 C01BD01 J01FA09
CYP-PGP inhibitors		J02AB02 J02AC02 J05AE10 J05AE08 J05AR14 J05AR15 J02AC01
Proton-pump inhibitors		A02BC
Vasodilators		C02DB C02DD C02DG C04 C05
Calcium		C07F C08 C09BB C09DB
Digoxin		C01AA05
Amiodarone		C01BD01
Thienopyridines		B01AC04 B01AC24 B01AC22
Phenprocoumon		B01AA04
Estimated glomerular filtration rate**	DNK35301; DNK35302; NPU04998	

*We identified subjects with hypertension from combination treatment with at least two of the following classes of antihypertensive drugs:
I. Alpha adrenergic blockers (C02A, C02B, C02C)
II. Non-loop diuretics (C02DA, C02L, C03A, C03B, C03D, C03E, C03X, C07C, C07D, C08G, C09BA, C09DA, C09XA52)
III. Vasodilators (C02DB, C02DD, C02DG, C04, C05)

IV. Beta blockers (C07)
V. Calcium channel blockers (C07F, C08, C09BB, C09DB)
VI. Renin-angiotensin system inhibitors (C09).

** Obtained from the Danish National Laboratory Registry

Supplemental Table 2: Baseline characteristics according to year of inclusion

Year of inclusion	**1997-2000**	**2001-2004**	**2005-2008**	**2009-2012**	**2013-2016**	**2017-2020**
N	29532	31543	30107	32170	22613	13017
sex % (N)	53.7 (15869)	53.7 (16951)	53.6 (16143)	52.2 (16802)	48.6 (10985)	45.8 (5964)
Age, median (IQR)	79.0 (72.0-85.0)	79.0 (71.0-85.0)	79.0 (70.0-86.0)	78.0 (69.0-85.0)	77.0 (69.0-84.0)	75.0 (69.0-83.0)
CHA_2DS_2-VA, median (IQR)	3.0 (2.0-4.0)	3.0 (2.0-4.0)	3.0 (2.0-4.0)	3.0 (2.0-4.0)	3.0 (2.0-4.0)	3.0 (2.0-4.0)
HASBLED, median (IQR)	2.0 (1.0-3.0)	2.0 (1.0-3.0)	2.0 (2.0-3.0)	2.0 (2.0-3.0)	2.0 (2.0-3.0)	2.0 (1.0-3.0)
Heart failure	29.7 (8768)	29.2 (9210)	27.5 (8270)	25.9 (8318)	23.9 (5394)	19.8 (2576)
Hypertension	35.5 (10483)	44.8 (14121)	54.1 (16295)	59.6 (19162)	60.2 (13612)	54.5 (7099)
Diabetes	11.5 (3404)	13.3 (4197)	13.8 (4148)	16.2 (5199)	18.1 (4102)	18.2 (2372)
Prior stroke	18.7 (5515)	20.0 (6296)	20.4 (6128)	19.8 (6366)	21.4 (4828)	21.0 (2730)
Vascular disease	14.1 (4173)	19.4 (6134)	20.2 (6083)	20.4 (6566)	20.6 (4650)	17.4 (2269)
Prior bleeding	9.0 (2655)	13.2 (4158)	15.9 (4791)	16.8 (5414)	19.1 (4322)	17.8 (2322)
Myocardial infarction	10.2 (3003)	14.4 (4552)	14.7 (4422)	14.7 (4718)	14.3 (3223)	11.5 (1491)
Ischemic heart disease	28.4 (8395)	32.3 (10190)	30.6 (9222)	29.9 (9627)	29.5 (6672)	24.3 (3165)
Hyperlipidemia	2.3 (688)	5.8 (1843)	10.6 (3183)	14.7 (4728)	19.2 (4336)	18.0 (2343)
PCI	0.7 (206)	2.5 (782)	4.9 (1482)	7.1 (2285)	8.7 (1967)	8.8 (1139)
CABG	1.6 (477)	2.4 (754)	3.1 (922)	3.5 (1122)	4.2 (952)	3.2 (414)
COPD	10.0 (2939)	12.7 (3995)	14.2 (4264)	15.4 (4957)	16.3 (3690)	14.6 (1904)
Alcohol	1.5 (456)	2.7 (849)	3.9 (1167)	5.0 (1620)	6.7 (1519)	7.1 (924)
Cancer	11.0 (3263)	15.2 (4792)	17.9 (5387)	21.5 (6901)	27.4 (6204)	31.2 (4063)
Alzheimer	0.6 (188)	1.3 (404)	2.1 (638)	2.4 (760)	2.5 (573)	2.2 (287)
Vascular dementia	0.6 (172)	1.0 (301)	1.3 (390)	1.0 (321)	0.9 (214)	0.7 (88)

Other dementia	2.9 (864)	3.9 (1232)	4.7 (1414)	4.1 (1312)	3.8 (863)	2.6 (338)
Renal disease	2.8 (837)	3.9 (1237)	4.8 (1438)	5.8 (1856)	7.4 (1674)	7.8 (1017)
Aspirin	37.3 (11009)	43.4 (13690)	46.2 (13918)	46.0 (14807)	38.2 (8629)	29.3 (3818)
Clopidogrel	0.2 (55)	2.1 (674)	4.2 (1258)	6.5 (2085)	12.7 (2872)	15.4 (2005)
Ticagrelor	0.0 (0)	0.0 (0)	0.0 (0)	0.3 (94)	1.6 (353)	1.3 (173)
Thienopyridines	0.2 (55)	2.1 (674)	4.2 (1258)	6.8 (2177)	14.2 (3202)	16.5 (2153)
Statins	3.7 (1092)	10.3 (3257)	23.2 (6986)	33.7 (10856)	38.4 (8689)	38.5 (5015)
NSAID	26.8 (7924)	29.3 (9253)	25.7 (7750)	23.4 (7539)	21.9 (4956)	19.0 (2470)
Beta-blockers	19.1 (5634)	26.1 (8238)	30.6 (9216)	33.6 (10815)	33.8 (7636)	31.2 (4062)
Calcium channel blockers	23.3 (6890)	23.3 (7352)	24.7 (7445)	28.7 (9240)	29.3 (6626)	28.2 (3672)
Loop diuretics	34.6 (10212)	32.4 (10207)	29.7 (8940)	27.0 (8684)	24.2 (5480)	20.7 (2698)
Non-loop diuretics	34.4 (10150)	37.9 (11940)	41.2 (12415)	41.2 (13241)	37.3 (8433)	30.8 (4014)
Vasodilators	4.9 (1453)	5.1 (1616)	5.1 (1534)	5.0 (1608)	4.6 (1035)	4.2 (552)
ACE/ARB inhibitors	19.2 (5665)	27.0 (8512)	35.8 (10790)	43.1 (13861)	45.4 (10263)	44.7 (5820)
Digoxin	27.7 (8184)	19.4 (6115)	11.8 (3549)	6.8 (2193)	3.9 (885)	2.1 (271)
Amiodarone	0.6 (164)	0.8 (251)	0.9 (258)	0.8 (248)	0.9 (201)	0.9 (120)

Supplemental Table 3: Medical treatment at baseline

Aspirin	42.9 (32701)	40.1 (33170)	41.4 (65871)
Clopidogrel	6.5 (4975)	4.8 (3974)	5.6 (8949)
Ticagrelor	0.6 (426)	0.2 (194)	0.4 (620)
Thienopyridines	7.0 (5375)	5.0 (4144)	6.0 (9519)
Statins	26.3 (20035)	19.2 (15860)	22.6 (35895)
NSAID	24.3 (18561)	25.8 (21331)	25.1 (39892)
Beta-blockers	28.5 (21742)	28.8 (23859)	28.7 (45601)
Calcium channel blockers	25.2 (19223)	26.6 (22002)	25.9 (41225)
Loop diuretics	26.9 (20480)	31.1 (25741)	29.1 (46221)
Non-loop diuretics	32.8 (24981)	42.6 (35212)	37.9 (60193)
Vasodilators	4.0 (3043)	5.7 (4755)	4.9 (7798)
ACE/ARB inhibitors	36.2 (27618)	33.0 (27293)	34.5 (54911)
Digoxin	11.9 (9083)	14.6 (12114)	13.3 (21197)
Amiodarone	1.1 (842)	0.5 (400)	0.8 (1242)

NSAID: Non-steroidal anti-inflammatory drugs. ACE: Angiotensin-converting enzyme. ARB: Angiotensin receptor blocker. AF: Atrial fibrillation.

Supplemental Table 4: One-year risk (%) of stroke stratified by sex, CHA_2DS_2-VA score level, and year of inclusion.

CHA_2DS_2-VA	Score 1		Score 2		Score 3		Score 4		Score 5+	
Sex	Males	Females	Males	Females	Males	Females	Males	Females	Males	Females
1997-2000	1.7	2.2	2.8	3.8	3.7	5.0	9.1	9.2	11.2	13.5
2001-2004	1.4	1.4	2.8	3.9	4.7	5.0	6.9	7.8	10.0	11.7
2005-2008	1.6	1.3	2.8	3.1	3.8	4.2	5.6	6.4	8.0	9.7
2009-2012	1.5	1.3	2.7	2.8	3.4	4.2	5.3	5.5	7.5	9.6
2013-2016	0.8	1.2	2.1	2.1	3.2	3.3	5.8	5.2	6.9	8.6
2017-2020	1.3	1.0	3.0	2.9	3.5	3.7	5.9	6.7	8.5	8.6

Supplemental Figure 1: All-cause mortality at 1-year follow-up for male and female patients with AF, n= 158,982.

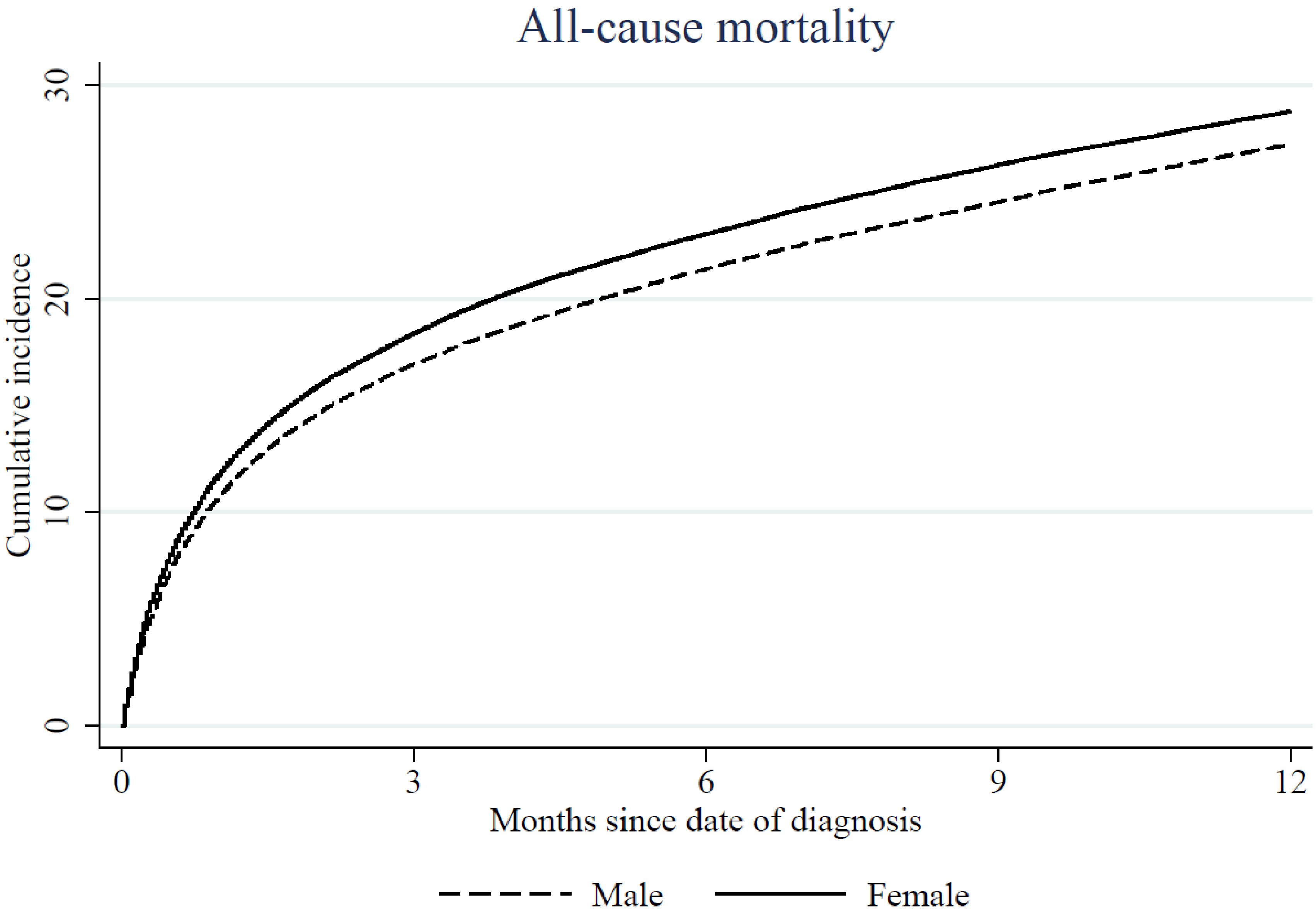

Supplement: Supplementary file 1 — Supplementary Information [file 41467_2024_51193_MOESM1_ESM.pdf]
